# Microbial Mats of the McMurdo Dry Valleys, Antarctica: Oases of Biological Activity in a Very Cold Desert

**DOI:** 10.3389/fmicb.2020.537960

**Published:** 2020-10-27

**Authors:** Jill A. Sohm, Thomas D. Niederberger, Alexander E. Parker, Joëlle Tirindelli, Troy Gunderson, Stephen Craig Cary, Douglas G. Capone, Edward J. Carpenter

**Affiliations:** ^1^Department of Biological Sciences, Marine and Environmental Biology Section, University of Southern California, Los Angeles, CA, United States; ^2^Environmental Studies Program, University of Southern California, Los Angeles, CA, United States; ^3^College of Earth, Ocean, and Environment, University of Delaware, Lewes, DE, United States; ^4^Estuary and Ocean Science Center, San Francisco State University, Tiburon, CA, United States; ^5^Department of Sciences and Mathematics, California State University Maritime Academy, Vallejo, CA, United States; ^6^International Centre for Terrestrial Antarctic Research, University of Waikato, Hamilton, New Zealand

**Keywords:** Dry Valleys, cyanobacteria, microbial mats, meltwater streams, MATS, diazotrophs

## Abstract

Cyanobacterial mats in the Antarctic Dry Valleys are photosynthetic microbial ecosystems living at the extreme of conditions on Earth with respect to temperature, light, water and nutrient availability. They are metabolically active for about 8 weeks during the austral summer when temperatures briefly rise above freezing and glacial and lake melt waters are available. There is much to learn about the biogeochemical impact of mats in these environments and the microbial communities associated with them. Our data demonstrate that these mats attain surprisingly high rates of carbon (CO_2_) and dinitrogen (N_2_) fixation when liquid water is available, in some cases comparable to rates in warmer temperate or tropical environments. C and N_2_ fixation in Dry Valley mats in turn substantially elevate dissolved organic C and inorganic N pools and thereby promote enhanced microbial secondary production. Moreover, the microbial community fingerprint of these mats is unique compared with the more ubiquitous dry soils that do not contain mats. Components of the heterotrophic microbiota may also contribute substantially to N inputs through N_2_ fixation.

## Introduction

The McMurdo Dry Valleys of Antarctica are thought to be the coldest, driest environments on Earth and the largest ice-free region on the continent (McKnight et al., 1999). Stretching over about 6500 km^2^, the Dry Valleys experience minimal precipitation, strong katabatic winds, and temperatures below −60°C during the austral winter (Fountain et al., 1999; Cary et al., 2010). These extreme conditions are the reason that the Dry Valleys were once considered to be unfavorable for life, with much of the dry soils thought to be sterile (Horowitz et al., 1972); in fact the Dry Valleys host a microbial community consisting of phylogenetically diverse bacteria, microalgae and fungi (McKnight et al., 1999; Wood et al., 2008; Cary et al., 2010; Dreesens et al., 2014). Microorganisms dominate the Dry Valley ecosystem (Cary et al., 2010), with a limited community of invertebrate grazers as the top of the food chain (Treonis et al., 1999). Climate in the Dry Valleys restricts the presence of vascular plants and larger animals, and thus limits the amount of organic material, making the Dry Valleys an extremely oligotrophic system. The presence and source of organic matter in the Dry Valleys is therefore a defining feature of the system with regards to life.

Organic matter in the Dry Valleys has three potential sources: resource legacies, current microbial communities and the nearby Ross Sea (Fountain et al., 1999; Moorhead et al., 1999; Hopkins et al., 2006). The Dry Valleys, while an arid cold desert for more than 10 million years, have gone through relative warm and cool periods during this time that altered the glaciers and hydrology, such that the Taylor Valley was sometimes covered by a lake (Lake Washburn) that was nearly 300 m deep and covered much of the valley floor (Fountain et al., 1999). The growth of primary producers at the edge of this historic lake left a “legacy” of organic matter. Today, primary producers grow in visible mats in more limited locations in the Valleys – along slow moving streams, under melting snow accumulations, in and around the edges of lakes, and in hypolithic and endolithic communities – and they may be distributed around the Valleys by strong winds when they dry up after summertime (Burkins et al., 2000; Wood et al., 2008).

While microbial signatures can be found throughout the Dry Valley soils, visible life tends to be concentrated near lakes, ponds and ephemeral streams (Paerl and Priscu, 1998; McKnight et al., 1999; Niederberger et al., 2012). This is due to the combination of available solar radiation and water from glacial and lake/pond ice melting during the brief austral summer, along with temperatures warm enough to allow for metabolic activity (McKnight et al., 2004). Water temperatures in the austral summer can range from zero to almost 9°C (Hawes et al., 1992). Wetted zones around lakes and ponds often support black or brown microbial mats dominated by *Nostoc spp*. (Niederberger et al., 2012) or orange/red mats dominated by *Phormidium* (Vincent et al., 1993). Black mat material may get entrained in the ice of the lakes in the form of aggregates that can fix CO_2_ and N_2_ in a self-sustaining microbial community (Paerl and Priscu, 1998). The stream systems have multiple types of mats specific to different parts of the stream channel: orange, red and green mats are typically found in the main channel, with black mats relegated to the wetted regions of the stream banks (hyporheic zone) (Alger et al., 1997; McKnight et al., 1999, 2004). The orange, red and green mats are dominated by the non-N_2_ fixing cyanobacterium *Phormidium* (orange/red) and diatoms (green), while the black mats are made up of the N_2_ fixing cyanobacterium *Nostoc spp*. (Alger et al., 1997). The mats are not ubiquitous, however, they are most common in shallow gradient streams with stone pavements that have developed over the long term through freezing of saturated porous alluvium (Mcknight et al., 1998; Fountain et al., 1999). While the abundance and identity of cyanobacteria in these mats has long been known, recent studies have tended to focus on the diatoms in stream systems [for example, see Stanish et al. (2011)], and the bacterial communities associated with different colored mats (Kohler et al., 2016; Van Horn et al., 2016). Studies that quantify physiological rates and elemental cycling associated with these mats, as well as the activity of associated heterotrophic microbes, are limited. The goal of our research was to quantify CO_2_ and N_2_ fixation in wetted areas of the McMurdo Dry Valley and determine physical, nutrient and biological factors that are associate with these activities.

## Materials and Methods

### Study Site

**FIGURE 1:**
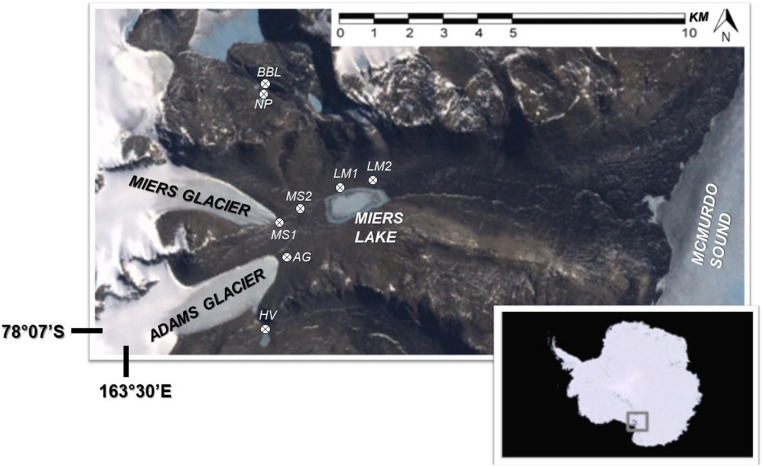
Study area of Miers Valley and adjacent sites, McMurdo Dry Valleys, Antarctica. Site designation: AG, Adams Glacier; BB, baby buddha lake; HV, Hidden Valley; LM 1 and 2, Lake Miers 1 and 2; MS 1 and 2, Miers Stream 1 and 2; NP, nostoc pond.

**FIGURE 2:**
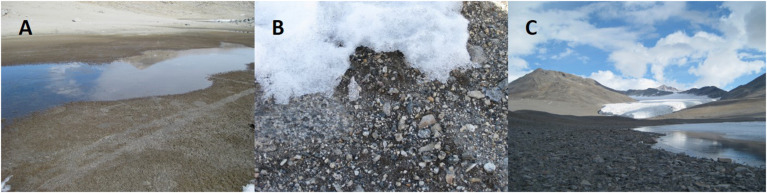
Images of mat environments in the Miers Valley and adjacent sites. **(A)** baby buddha lake (BB), and example of mats around a small pond; **(B)** mats under a patch of snow next to Miers Stream (MS1 in Figure 1); **(C)** south side of Lake Miers, where mats were also found.

In our study, conducted in January and December 2009, N_2_ and CO_2_ fixation activity, bacterial activity, and nutrient concentrations in lakes/ponds and pore waters were investigated in association with cyanobacterial mats located in, and at sites adjacent to, the Miers Valley, one of the smaller (11 km long) and southerly of the McMurdo Dry Valleys (Figure 1). The Miers Valley is characterized by two glaciers – the Adams and the Miers – at the west end that feed two glacial melt water streams during austral summer. When temperatures are particularly high and water flow increases, the large region in between these streams also becomes wetted due to stream overflow. These streams feed into Lake Miers (LM), which has an outflow toward McMurdo Sound. At higher elevations to the north and south of this valley are smaller ponds including two that we refer to as nostoc pond (NP) and baby buddha lake (BB). The lake and ponds are covered with ice year-round, but in the summer the ice melts at the edges and creates a moat around the ice and a wetted area around the lake/pond. Images of selected sites can be seen in Figure 2. For this study, a total of 8 wetted locations next to stream, lakes, and ponds were sampled over 2 years. Some of these locations were sampled in both year one and two of the study.

At each sampling location, transects from the wet to dry soil were sampled. A transect consisted of 3 or 4 sampling points that included one in the water, one just adjacent to the water and one in the dry soil nearby the water source. In cases where the wetted area was wider, a fourth point was sampled in the wetted area along the transect. A map of the sampling locations can be seen in Figure 1. Further details on sampling sites and strategies can be found in Niederberger et al. (2012).

### N_2_ Fixation

Dinitrogen fixation was measured in the field as nitrogenase activity using the acetylene (C_2_H_2_) reduction assay (Capone, 1993), with some modifications. A cut off syringe was used to collect the top 1 cm of mat and mineral sands or mineral sands alone and 4 or 3 (years 1 and 2, respectively) of these 1 cm “plugs” were placed into a 27 ml glass serum vial. The serum vial was sealed, 2 ml of C_2_H_2_ was added, and the increase in ethylene (C_2_H_4_) was assayed over ∼8 h using a Shimadzu Mini-2 gas chromatograph fitted with a 6′ HayeSep A column. Each sample was run in triplicate except for the dry adjacent sands, of which a single sample was run (as these were always zero activity). The rate of N_2_ fixation was calculated over the linear period of C_2_H_4_ increase, using the standard calculation and a conversion factor of 3:1 mole C_2_H_2_ fixed per mol N_2_ fixed (Capone, 1993).

### CO_2_ Fixation

Carbon fixation was determined with the uptake of ^13^C-bicarbonate into organic matter. Three 1-cm plugs of sample from a site were placed into a 27 ml serum vial. Water from adjacent to the site was used to fill the bottle, the bottle was sealed and all air bubbles removed. 10 μl of 0.233 M ^13^C bicarbonate was added and the samples were incubated in ambient conditions for ∼24 h before the isotope uptake was stopped by placing the sample in a 50 ml centrifuge tube, centrifuging at low speed, then pouring off the overlying water. The sample was then washed three times in this manner with water from adjacent to the site and then stored in the dark and cold before the samples could be placed in the freezer at McMurdo Station. Samples were dried at McMurdo Station and then homogenized with a mortar and pestle and weighed at the University of Southern California. d^13^C values were determined using continuous flow mass spectrometry by the UC Davis Stable Isotope Facility. The CO_2_ fixation rate was calculated using the equations of Montoya et al. (1996) modified for sediment samples and using the natural abundance of samples from each site as the initial value (or an average of similar sites where no natural abundance data existed) and average dissolved inorganic C (DIC) concentrations for stream or pond/lake water.

### Bacterial Activity (Thymidine Uptake)

Bacterial activity was measured as the uptake of thymidine into cells. Two 1-cm plugs of mat were placed into sterile Whirl pak bags with 2 ml of water from adjacent to the sample site and homogenized. ^3^H-thymidine was added to a concentration of 20 nM and samples were incubated for 2–6 h in ambient conditions. Activity was terminated by adding 1 ml formalin. Samples were then filtered onto 0.2 mm cellulose nitrate filters using a Hoeffer filtration unit, rinsed three times with 1 mM cold thymidine, and then placed into a 15 ml centrifuge tube and stored in the cold and dark until transport back to McMurdo Station. Samples were run in triplicate and a killed control that had formalin added before the isotope was performed for each set of replicates.

At McMurdo Station, microbial mat cells were lysed in 5 ml of a 0.3N NaOH/0.1% SDS/25 mM EDTA solution added to the sediments at 25°C for 12 h (Findlay, 1993). After extraction and a brief centrifugation (2000 × *g*, 5 min), 0.5 ml of the supernatant was placed in a scintillation vial with scintillation cocktail and the activity was determined on a Beckman LS6000 liquid scintillation counter. This extract contains labeled DNA, protein, and RNA. Thymidine uptake was then calculated from the disintegrations per minute (DPM) in each sample and the incubation time, accounting for sediment volume and activity in the killed control.

### Inorganic and Organic Nutrients

Samples for pore water NO_3_^–^, PO_4_^3–^, Si(OH)_4_, NH_4_^+^, dissolved organic C (DOC) and total dissolved N (TDN) were taken in wetted zones by removing the top layer of sediment until the pore water was revealed and pumping out the water into a clean flask. Pore water was filtered through combusted GF/F filters and frozen in appropriate vessels for later analysis. Nutrient analysis was performed on a Bran and Luebbe AutoAnalyzer II with MT-19 manifold chemistry module according to Whitledge et al. (1981) and Bran and Luebbe Method G-172-96, Method G-175-96 and G-177-96. For NO_3_^–^ + NO_2_^–^, PO_4_^3–^ and Si(OH)_4_, respectively. Samples for NH_4_ were analyzed according to Solorzano (1969) and DOC and TDN were determined by high temperature combustion using a Shimadzu TOC-V (Sharp et al., 2004). Dissolved organic N (DON) was calculated as the difference between TDN and the sum of dissolved inorganic N (NO_3_^–^ + NO_2_^–^, and NH_4_^+^).

Samples for dissolved inorganic C (DIC) concentration were gently poured into 20 ml borosilicate scintillation vials, preserved with 200 μl 5% (wt:vol) mercuric chloride and stored in the dark at 4°C until analysis (Sharp, 2002). Prior to analysis, samples were brought to room temperature and analyzed using a Monterey Bay Research Institute-clone DIC analyzer with acid-sparging and non-dispersive infrared analysis [LI-COR CO_2_ Analyzer, Model LI-6252; (Friederich et al., 2002)]. DIC determinations were made from a single point calibration using certified reference material (A. Dickson at the Oceanic Carbon Dioxide Quality Control, Marine Physical Laboratory at Scripps Institution of Oceanography, UCSD) and were based on triplicate 1.5 mL injections for each determination.

### Other Sediment Analyses

Gravimetric water content was determined by taking the upper 1 cc of soil and placing it in a 15 ml centrifuge tube for later analysis. At McMurdo station, the sample tubes were weighed, DI water was added to 3 ml, and the tubes were weighed again before drying at 65°C. After the dry weight was determined, gravimetric water content was calculated as the volume of water in the sediment divided by the wet weight of the sediment. Chlorophyll was extracted in acetone for 24 h at −20°C from a separate 1 cc surface soil sample and the fluorescence read on a Turner 10AU field fluorometer.

### Estimating Total Area of Microbial Mats Around Lake Miers

In order to estimate the coverage of microbial mats around Lake Miers, the perimeter of the lake was walked while observing presence of the mats. Using a surveyors wheel, we measured the length of the areas containing mats. Each time a multiple of 100 m was reached on the wheel, the width of the mat area was measured and recorded. On the north side of the lake, 667 m of mats were recorded, with an average width of 0.44 m (SD 0.28 m) while mats were recorded along 1129 m of the south side of the lake, with an average width of 1.88 m (SD 0.96 m). The estimate for total area of coverage was made by multiplying length of mats by average width for each side of the lake.

### Microbial Community Diversity Analyses

Bacterial t-RFLP analyses were used as a community fingerprinting tool. While this method is not commonly used today, it is useful for tracking overall changes in community diversity and comparing this between disparate environments or treatments. As such, t-RFLP is an appropriate method to address our goal to compare the effects N_2_ and CO_2_ fixing microbial mats and other environmental parameters on overall community composition. Bacterial t-RFLP were undertaken as previously described (Niederberger et al., 2012, 2015). For each sample, all peaks (OTUs) that were less than 0.5% of the total peak height were removed, as they were likely to be noise with this method. Peak heights for each OTU were then normalized to the total peak height, and a CCA was performed in the package “vegan” in R (Oksanen et al., 2019), using the following as constraining variables: the N_2_ fixation rate (N fix), the presence of a visible mat (Mat), whether the sample was wet or dry (Wet), and location within, or adjacent to, Miers Valley (Miers). These variables were chosen based on anosim and envfit results in vegan (Ramette, 2007), and subsequent ANOVA of the CCA showed all four variables to be significant (*p* < 0.02).

### Other Statistical Analyses

Averages of the data are reported with standard error (S.E.). *T*-tests (unequal variance assumed) were performed using PSPP software to compare soil properties and activity in stream, lake, and dry soils and to compare nutrients in water and porewater. Correlation analysis (Pearson’s *r*) of soil and porewater properties was carried out in *R* on square-root normalized data and visualized using the package “corrgram.”

## Results and Discussion

### Rates of N_2_ and CO_2_ Fixation in and Adjacent to the Miers Valley

**FIGURE 3:**
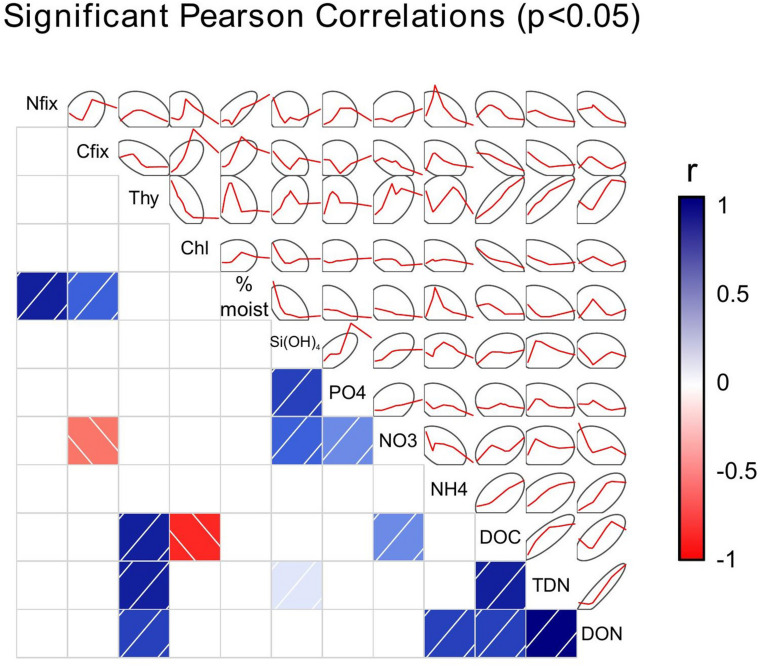
Correlogram of the Pearson’s r correlation matrix between properties measured. Correlation was carried out on square root normalized data. The upper panel shows the smoothed lines of the data comparisons with the 95% confidence interval ellipse while the lower panel shows the *r* value for the correlation between each pair of measurements, with only those with significance *p* < 0.05 displayed (two tailed, unequal variance).

Dinitrogen and CO_2_ fixation rates were highly variable in the Miers Valley and adjacent areas and ranged from 0 to 10.9 nmol N cm^–3^ h^–1^ and 0 to 7.05 mmol C cm^–3^ h^–1^, respectively (Table 1 and Figure 3); the N_2_ fixation rates are of the same order of magnitude reported for two samples in a previous Dry Valleys study (Howard-Williams et al., 1989).

**TABLE 1:** Biological activity rate measurements in wetted and adjacent dry soils.

Site	N_2_ Fix			C fix			Thy		
					
Name	Average	SE	N	Average	SE	N	Average	SE	N
	nmol N cm^3^ h^−1^			nmol C cm^3^ h^−1^			pmol cm^3^ h^−1^		
**Stream/Wetted**									
MS1 Y1	1.90	0.52	6	ND	ND	0	ND	ND	0
MS2 Y1	0.19	0.10	6	14	2	6	10.37	2.64	3
MS1 Y2	1.84	0.43	6	30	30	4	4.72	2.63	6
Adams in Y2	3.65	1.37	3	ND	ND	0	5.29	1.73	3
ALL DATA	1.64	0.35	21	21	11	10	6.28	1.59	12
**Pond/Lake**									
LM1 Y1	1.31	0.43	9	110	118	7	20.66	3.76	6
LM2 Y1	1.04	0.15	9	350	111	7	36.31	5.46	3
NP Y1	3.55	0.71	9	ND	ND	0	ND	ND	0
NP Y2	10.87	3.44	6	7054	1669	4	7.80	1.67	6
BBL	1.27	0.42	9	212	112	9	ND	ND	0
LM1 Y2	0.76	0.13	6	901	279	4	6.63	0.61	6
HV	1.54	0.43	9	5322	774	9	0.00	0.00	3
LM2 Y2	1.30	0.52	9	ND	ND	0	8.17	2.42	6
ALL DATA	2.42	0.47	66	2121	479	40	12.28	2.11	30
**Dry**									
MS1Y1	0.46	0.16	3	0	0	3	ND	ND	0
MS2Y1	0.00	0.00	3	0	0	3	ND	ND	0
LM1	0.00	0.00	3	1		2	ND	ND	0
LM2	0.00	0.00	3	ND	ND	0	ND	ND	0
NP	0.00	0.00	3	ND	ND	0	ND	ND	0
BBL	0.00	0.00	3	5		1	ND	ND	0
NP Y2	0.03	0.01	3	ND	ND	0	ND	ND	0
LM 1 Y2	0.00	0.00	3	ND	ND	0	ND	ND	0
AG out	2.30	1.17	3	ND	ND	0	1.16	0.22	3
LM2 Y2	0.03	0.03	3	ND	ND	0	3.21	0.50	3
ALL DATA	0.28	0.16	30	0.80	0.71	9	2.19	0.41	6

*Values were averaged for both field seasons. Data summarized from Supplementary Tables S1A–C for the respective environments. Standard error (S.E.) and *n* value shown in parentheses. MS, Miers Stream, LM, Lake Miers, NP, nostoc pond, BB, baby buddha lake, HV, Hidden Valley, AG, Adams Glacier.*

A general pattern was observed across three habitat types sampled, lake/ponds, stream, and dry soil, with highest rates for N_2_, CO_2_, and bacterial production found in lake/ponds, followed by streams and finally dry soil. Specifically, N_2_ fixation rates were 50% higher in lake/pond habitats when compared to streams, while rates of N_2_ fixation in dry soils were lower still, only 20% of those observed in streams. Similarly, CO_2_ fixation in lake/pond habitats was 100-fold higher than streams, while CO_2_ fixation in dry soil habitats was 3% of values measured in streams. Finally, bacterial production was nearly 2-fold higher in lake/pond habitats compared to streams, while dry soil bacterial production rates were 35% of those measured in streams.

We observed variability in rate measurements within habitat types. For example, within lake/pond habitats, nostoc pond (NP) and Hidden Valley (HV) supported very high rates of CO_2_ fixation compared to rates measured at Lake Miers. Similarly, N_2_ fixation varied substantially within habitats; N_2_ fixation at MS 1 and MS 2 sites, both stream habitats, were an order of magnitude different. We also observed temporal (interannual) variability in rate processes, with samples at nostoc pond varying 3-fold between January and December 2009.

The wide range in N_2_ and CO_2_ fixation activity in the Miers Valley is mainly due to differences in activity between wetted and dry soils (which in turn is because overall biomass is lower (Niederberger et al., 2012) and mats are not visible in dry soils), but activity also covered a wide range within wetted sites between sampling locations (Table 1 and Figure 3). Wetted soils next to streams and lakes/ponds were characterized by significantly higher N_2_ fixation rates than adjacent dry soils (*p* = 0.024 and *p* < 0.001, respectively), while only lake/pond soils showed higher CO_2_ fixation rates than dry soils (*p* = 0.21, Table 1). In fact, dry soil and stream samples had CO_2_ fixation rates that were three orders of magnitude lower than lake soils, although the average value for streams and dry soils is based on a smaller number of samples than for lake/pond soils (Table 1).

Remarkably, N_2_ and CO_2_ fixation activity in the Miers Valley was similar to activity seen in temperate ecosystems, along streambeds and in intertidal regions (Table 2). While the Dry Valley system seems inhospitable to life, it does have one of the most important requirements – liquid water – albeit only in the summertime and only in localized areas. As seen here, the presence of water by itself is not the only requirement for mat growth, but it can make it possible. While there is not a long term weather station in the Miers Valley, the summertime temperatures in the nearby and also coastal Taylor Valley are above freezing on many days (Doran et al., 2002), and some microbes and cyanobacteria have adaptations for maintaining activity in cold temperatures, surviving daily freeze-thaw cycles, and surviving wintertime dormancy (Quesada and Vincent, 2012; De Maayer et al., 2014; Gao et al., 2020). It is important to note here that the coastal Valleys are warmer and wetter than some of the other Dry Valleys (Doran et al., 2002; Goordial et al., 2016) so these results may not be broadly applicable across the entire system of approximately 15 valleys.

**TABLE 2:** N_2_ and CO_2_ fixation rates in microbial mats from Antarctic locations and fresh water and marine locations in warmer climates.

Location	N_2_ fixation	CO_2_ fixation	Comment	Study
	(nmol N cm^–^^3^ h^–^^1^)	(μmol C cm^–^^3^ h^–^^1^)		
**Miers Valley area, Antarctica**				
Streams (mean ± s.e.)	1.64 (0.35)	0.021 (0.01)		
Ponds: (mean ± s.e.)	2.42 (0.47)	2.121 (0.28)		This Study,
South Shetland Islands, Antarctica				Fernández-Valiente et al., 2007
Soil	0.14	0.20	mats	
Pond	1.42	0.23		
Taylor Valley stream, Antarctica	3.75		rewetted stream bed	McKnight et al., 2007
Sawtooth Lake-Stream District, Idaho	<0.4		periphyton	Marcarelli and Wurtsbaugh, 2006
Rocky Creek, CA (annual mean)	0.15		*Nostoc*	Horne and Carmiggelt, 1975
Intertidal mat Beaufort, NC	6.8			Steppe and Paerl, 2005
Twin Cays, Belize	0–1.5 days,	1.5–2.0 night		Joye and Lee, 2004
Tomales Bay, CA, United States	12.5			Joye and Paerl, 1993
Island of Mellum, Germany	2.9–6.0			Stal et al., 1984
Coral Reefs (average 7 studies)	1.8	54		Capone, 1988
Rocky shore (average 2 studies)	2.9	2.1		Capone, 1988
Salt marsh (average 4 studies)	1.8	16.4		Capone, 1988
Sippewissett Marsh, Cape Cod, MA, United States	2.11			Van Raalte et al., 1974
Coastal, NC	5.95			Bebout et al., 1987
Desert Stream, AZ	12.5	Epilithic		Grimm and Petrone, 1997
Desert Stream, AZ	24.7		Cyanobacteria	Grimm and Petrone, 1997
Desert Stream, AZ	51.4		*Anabaena*	Grimm and Petrone, 1997
Rocky Shore, United Kingdom	2.04			Stewart, 1967
Coral Reef, Marshall Isl.	56.8		*Calothrix*	Wiebe et al., 1975
Desert Crusts, Utah	up to 8.7^*a*^		*Collema* (lichen), *Nostoc Scytonema*,	Belnap, 2002
Desert Crusts^*b*^				Housman et al., 2006
Utah	0.14- 0.18	111–139		
New Mexico	0.05–0.18	97–242		

*a = assumed to 1 cm. b = cores collected to 3 cm.*

### Relationship Between N_2_ and CO_2_ Fixation

While it has tacitly been assumed that N_2_ fixation in microbial mats of the Dry Valleys is carried out solely by the cyanobacteria (Vincent and Howard-Williams, 1986), N_2_ and CO_2_ fixation were not correlated with each other in the areas measured in and adjacent to Miers Valley (Figures 3, 5). The decoupling of N_2_ and CO_2_ fixation may be explained by the fact that sulfate reducing bacteria were responsible for up to half of N_2_ fixation activity in Miers Valley samples (Niederberger et al., 2012). Additionally, CO_2_ fixation is likely also carried out by non-N_2_ fixing organisms like eukaryotic algae, as corroborated by phylogenetic analyses (Niederberger et al., 2016). The mechanisms and drivers that decouple N_2_ and CO_2_ fixation deserve further investigation.

### The Importance of Water for *Nostoc* Mats

Microbial mats containing the cyanobacterium *Nostoc commune* and other microbes were found in many of the wetted regions of the Miers Valley, including the melted moats around lakes and ponds, the hyporheic zone between slow-moving streams, and under slow-melting snow patches adjacent to streams. Mats were not seen in the wetted region on the banks of the fast moving streams. Thus, *Nostoc* mat formation appears to depend not only on the presence of liquid water, but water must be stagnant or slow-moving, to prevent washout of mat organisms that might otherwise be able to colonize wetted regions. In contrast to the more common *Nostoc* mats, the orange/red mats comprised of *Phormidium* (Vincent et al., 1993), were found in only one of the ponds that we surveyed, adjacent to the Miers Valley (nostoc pond). This is in contrast to the Taylor Valley, where *Phormidium* has been observed commonly in the quickly-flowing stream channels (Alger et al., 1997; McKnight et al., 2004).

In addition to the association of microbial mats with wetted areas, within microbial mats, soil moisture content positively correlated with microbial activity. Both N_2_ fixation and CO_2_ fixation were positively correlated with% soil moisture, although the relationship was stronger and with a greater level of significance for N_2_ fixation (N_2_ fixation: *r* = 0.65, *p* < 0.001; CO_2_ fixation: *r* = 0.41, *p* = 0.031) (Figures 3, 5).

Desert soil crusts are similarly highly dependent on hydration for high levels of N_2_ fixation. Highest rates were found after precipitation event, but not if preceded by extended dry spells (Belnap, 2002).

### Porewater Nutrients

Nutrients in porewater showed correlations suggesting common origins. PO_4_^3–^ and Si(OH)_4_ and NO_3_^–^ were all significantly correlated with each other (*r* > 0.4, *p* < 0.02), which is expected because of the sources of these nutrients (Figure 3). PO_4_^3–^ and Si(OH)_4_ are part of rock minerals, and leach into the water through chemical weathering processes (Gooseff et al., 2002; Green et al., 2005). NO_3_^–^ is also leached from Dry Valley soils, but the ultimate source appears to be atmospheric deposition (Green et al., 2005; Michalski et al., 2005). Nonetheless, these nutrients are leached from the soils as streams flow in the summertime, and the weathering rates have been shown to be remarkably high for a cold, dry ecosystem (Gooseff et al., 2002; Green et al., 2005). Other than a significant correlation between Si(OH)_4_ and TDN and NO_3_^–^ and DOC, putatively leached nutrients were not correlated to nutrients that we would consider to be indicators of biological processes, including NH_4_^+^, DOC, DON or TDN. One interpretation of this is that abiotic nutrient sources are not driving the growth and activity of microbial mats that produce organic nutrients in the Miers Valley and surrounding area; more likely is that there is temporal mismatch in nutrient sources, production, and consumption that make it difficult to tease out the relationships with samples from a single point in time. Van Horn et al. (2016) encountered a similar result where stream chemical parameters were largely uncorrelated with mat diversity patterns in Dry Valley streams (focused largely on Taylor Valley). Here too this is likely indicative of both the issue of comparing measures that develop on different timescales but may also relate to the fact that mat development has many factors that influence it, including hydrological regime and other factors that were not measured. As would be expected, some of the nutrients associated with biological activity were correlated with each other: DOC and DON and DON and NH_4_^+^ both showed significant correlations with each other (*r* = 0.52, *p* = 0.013; *r* = 0.48, *p* = 0.039, respectively).

Porewater samples from the hyporheic zone around streams and lakes were enriched in DOC and TDN compared to overlying water from Miers Stream and Lake Miers (Table 3). Dissolved organic C in overlying waters averaged 29.2 μM, similar to that seen in the Taylor Valley (McKnight et al., 1993), but was an order of magnitude larger, 437 μM, in porewater (*p* < 0.001), suggesting that N_2_ and CO_2_ fixation that occurs within *Nostoc* mats contribute to pore water dissolved organic matter. This organic matter is likely to be an important nutrient source for microbes living in streams (see below), and others have shown that *Nostoc* mats are an important source of N for downstream mats in Dry Valleys streams, compared to glacial N sources (Kohler et al., 2018). Still, DOC and TDN concentrations were not directly correlated with CO_2_ or N_2_ fixation rates in our study, respectively, suggesting a decoupling of production of dissolved organic matter within mats and their accumulation in porewater (Figure 3). This is not surprising given that our activity measurements are an instantaneous measure of production, whereas porewater concentrations reflect both production and consumption, both occurring over longer time scales.

**TABLE 3:** Inorganic and organic nutrient concentrations in pore water versus stream/lake water (overlying water) from seven sites along the Miers Stream, and water near the inlet and outlet of Lake Miers.

Nutrient	Overlying water	Porewater
	µM	µM
Nitrate	3.67 (0.25; 11)	14.1 (3.0; 26)
Phosphate	0.24 (0.03; 11)	0.72 (0.14; 26)
Silicate	0.07 (0.05)	113 (14; 26)
DOC	29.2 (7.8; 12)	437 (65; 31)
TDN	4.52 (0.21; 12)	46.4 (6.0; 23)

*The pore water values are averaged from all samples where nutrients were determined. Standard error (S.E.) and sample number (n) shown in parentheses. Pairwise comparison between overlying water and porewater for each nutrient was significantly different (*p* < 0.003 for all nutrients, teo tailed, unequal variances).*

### Heterotrophic Bacterial Production and Community Drivers

**FIGURE 4:**
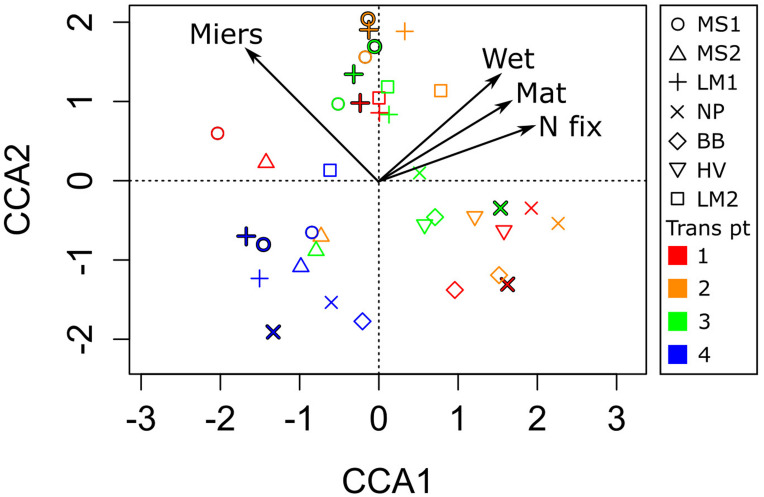
Canonical Correspondence Analysis (CCA) of microbial communities in and around the Miers Valley, as determined with t-RFLP and constrained with N_2_ fixation rates (Nfix), presence of moisture (Wet), presence of microbial mat (Mat), and location within or adjacent to Miers Valley (Miers). Each sampling location is shown with a different symbol, and the point along the sampling transect is designated by color and listed under “Trans pt” in the legend. Red/1 was a sample from in the water source, out to blue/4 as dry soil with no visible mat. In some cases, locations were sampled in both year 1 and year 2 of the study; in these cases, year 2 samples are outlined in black.

Thymidine uptake was enhanced in both wetted soils associated with streams and lake/pond sites compared to dry soils. In the case of the lake/pond soils, this difference amounted to a 6-fold increase (*p* = 0.012, Table 1). Bacterial production may be stimulated by organic matter substrate enrichment in porewater as thymidine uptake was significantly correlated with porewater DOC (*r* = 0.9, *p* < 0.001), DON and TDN (Figure 3). Additionally, the community composition of bacteria associated with the mats was distinct from the community in arid soils (Figure 4). Canonical Correspondence Analysis of 16S t-RFLP data showed that the four environmental variables investigated were able to constrain only 17% of the community variability. The dry soil communities from all sampling locations, which were collected at the fourth point on the transect at each location (transect pt. 4 in Figure 4), were similar to each other and different from communities associated with wet soils, mats, and high N_2_ fixation rate sites. Moisture has been shown by others to be a significant driver of bacterial communities in the Dry Valleys; species diversity is far lower in arid soils, and the community structure is clearly different compared to wet soils (Lee et al., 2018). However, sampling location also played a role in determining community similarity in our study, as the samples collected in and just adjacent to water sources in the Miers Valley ordinated together, while samples collected in and just adjacent to water sources from the Miers Valley adjacent locations (both north or south) ordinated together (Figure 4). These results agree with the work of Stanish et al. (2011) showing that Dry Valley microbial mats support specific bacterial taxa and that specific bacterial and cyanobacterial taxa co-occurred with specific diatom taxa in Dry Valley streams. Similar types of mats in the Dry Valleys (based on color) are shown to contain different microbial communities when comparing across streams, meaning the different types are not by themselves a consistent habitat type and the local physical, chemical, and hydrological conditions are important factors (Kohler et al., 2016). Our data cannot be used to determine these specific relationships in our mat samples, however, our more recent study of the phylogeny of N_2_ fixers has established some of these relationships and has corroborated the importance of heterotrophic N_2_ fixers (Coyne et al., 2020). Community composition was similar in samples that were taken from within one location in both January and December 2009, indicating that bacterial communities are consistent from year to year.

**FIGURE 5:**
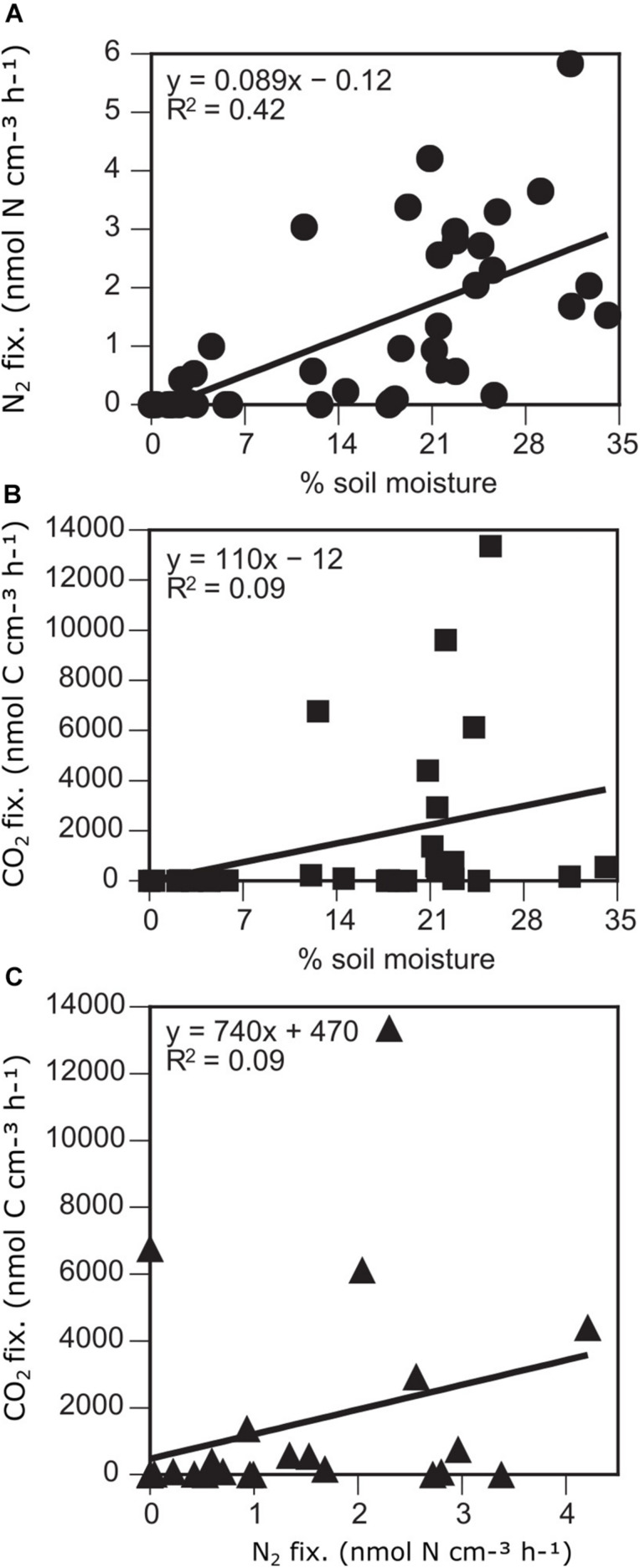
N_2_ **(A)** and CO2 **(B)** fixation versus soil moisture and N_2_ versus C fixation **(C)** in soils of the Miers Valley and adjacent sites.

### CO_2_ and N_2_ Fixation Rates at the Ecosystem Scale

During the second field season, we surveyed the extent of microbial mats around Lake Miers in order to make an estimate of its total contribution to N and C budgets in the Miers Valley. Attention focused on the lake because mats along streams in Miers Valley, while present, were patchy and relatively infrequent compared to mats around the lake. The mats covered a linear total of ∼1800 m of the perimeter of the lake, with an average width of the mat of 0.44 m on the north side of the lake and 1.8 m on the lake’s south side. We identified two mat types, a black mossy mat found closer to the water that covered 1/6 of the ground that it occupied, and a patchy brown mat farther from the water that covered approximately 1/12 of the ground. The remainder of the area was bare ground and rocks that were interspersed between mats. Using these estimates and the values for CO_2_ and N_2_ fixation in the two types of mats at lake sites, and a total of 100 active days (Frederick and Liao, 2005), we estimate that the microbial mats around the lake contribute approximately 75 kg C yr^–1^ and 0.3 kg N yr^–1^ to Miers Valley. While this estimate does not include all mats in Miers Valley, much of the mats observed there were around the lake, and thus this should act as a reasonable estimate of C and N inputs from summer wetted areas. This estimate also excludes CO_2_ fixation within the lake itself. Based on evidence from Taylor Valley, Miers Lake is likely to be net heterotrophic annually, despite photo- and chemo-autotrophic CO_2_ fixation occurring in summer (Vick-Majors and Priscu, 2019). As shown in this study, dry soils, which make up the bulk of the landscape in the McMurdo Dry Valleys, are not major sites of CO_2_ and N_2_ fixation. There are, however, cryptic microbial communities (hypoliths and endoliths) found there. Hypolithic N_2_ fixation across three small valleys (including the Miers, Marshal, and Garwood Valleys) has been estimated at 0.38 kg N yr^–1^ (Cowan et al., 2011). Nitrogenase activity has been detected in endolithic samples (Banerjee and Verma, 2009), and both prokaryotic and eukaryotic primary producers are part of endolithic communities (De Los Ríos et al., 2014), but the contribution to N and C budgets across entire valley systems is unclear. Taken together, this indicates that the lake, streams, and cryptic microbial communities are contributing substantial modern C and N to the Miers Valley. How does this compare to legacy and marine contributions? Legacy C and N estimates are difficult to find, but Moorhead et al. (1999) attempted to use the age and nutrient concentrations of Lake Hoare bottom waters to estimate the yearly contributions necessary to account for their presence. They calculated that approximately 0.37 kg N y^–1^ of input could account for the current N accumulation in Lake Hoare (in the nearby, but much larger, Taylor Valley). Burkins et al. (2000) expanded on this model using stable isotopic signatures of organic matter throughout the Taylor Valley; they propose that previous high lake levels supported microbial mat growth across a wide swath of the Taylor Valley, stranding it in place as the lake shrank, and this material, plus glacial till deposition at that same time support much of the needs of heterotrophs, rather than transport of modern material. Marine deposition appears important in areas closer to the ocean, while endolithic sources are important at higher elevations. It is unclear, however, if this model is applicable in other Dry Valleys, specifically if lakes in other Valleys were expanded during previous periods in order to leave these legacies. Regardless, the alignment of historical N input to Lake Hoare, endolithic N_2_ fixation (Cowan et al., 2011) and N input from mats surrounding Miers Lake (this study) suggest modern nutrient sources are important in Miers Valley. C turnover times in the 10s of years in the Dry Valleys support the importance of modern organic matter in supporting heterotrophic organisms in this system (e.g., Barrett et al., 2006).

## Conclusion

This work demonstrates that water can bring extreme microbial activity to the very oligotrophic Dry Valleys, and that activity in these mats can stimulate other microbial activity, and contribute important nutrients to a very nutrient poor landscape. While the Dry Valley system appears barren, there are summertime oases of activity that promote unique populations of associated bacteria.

## Data Availability Statement

The datasets generated for this study are available on request to the corresponding author.

## Author Contributions

SC, DC, EC, JS, and TN were responsible for the experimental design. JS, TN, AP, JT, TG, and DC carried out the field work and laboratory analysis. JS and AP analyzed the data. JS, AP, SC, DC, and EC wrote the manuscript. All authors contributed to the article and approved the submitted version.

## Conflict of Interest

The authors declare that the research was conducted in the absence of any commercial or financial relationships that could be construed as a potential conflict of interest.

## Supplementary Material

Supplementary Table 1A N_2_ fixation, CO_2_ uptake and thymidine incorporation for stream and wetted.

Supplementary Table 1B N_2_ fixation, CO_2_ uptake and thymidine incorporation for ponds and lake sites.

Supplementary Table 1C N_2_ fixation, CO_2_ uptake and thymidine incorporation for dry soil sites.
