# Supplementary material for: Microbial Mats of the McMurdo Dry Valleys, Antarctica: Oases of Biological Activity in a Very Cold Desert
Source: Front Microbiol. 2020 Oct 27;11:537960. doi: 10.3389/fmicb.2020.537960 (PMC7654227; doi:10.3389/fmicb.2020.537960)
Supplement: Supplementary Table 1A — N2 fixation, CO2 uptake and thymidine incorporation for stream and wetted. [file Table_1.pdf]

Supplemental Table 1a

Stream / Wetted		N2 fix	C fix	Thy		N2 Fix			C fix			Thy		
Site	Pt	nmol cc h	nmol cc h	pmol cm3 h		nmol cm3 h			nmol cm3 h			pmol cm3 h		
Name	#					average	SE	N	average	SE	N	average	SE	N
MS1 Y1	2	1.353	ND	ND	MS1 Y1	1.90	0.52	6	ND	ND	0	ND	ND	0
MS1 Y1	2	1.008	ND	ND										
MS1 Y1	2	0.237	ND	ND										
MS1 Y1	3	2.235	ND	ND										
MS1 Y1	3	3.755	ND	ND										
MS1 Y1	3	2.822	ND	ND										
MS2Y1	2	0	14.9	5.44	MS2Y1	0.19	0.10	6	14.18	1.70	6	10.37	2.64	3
MS2Y1	2	0	11.6	14.49										
MS2Y1	2	0	11.0	11.18										
MS2 Y1	3	0.208	9.9	ND										
MS2 Y1	3	0.342	20.8	ND										
MS2 Y1	3	0.564	16.8	ND										
MS1 Y2	1	1.261	120.6	0.26	MS1 Y2	1.84	0.43	6	30.14	30.14	4	4.72	2.63	6
MS1 Y2	1	1.361	0	0.00										
MS1 Y2	1	0.243		0.15										
MS1 Y2	2	2.905	0	12.50										
MS1 Y2	2	2.855	0	1.99										
MS1 Y2	2	2.389		13.45										
Adams in Y2	1	2.998	ND	5.15	Adams in Y2	3.65	1.37	3	ND	ND	0	5.29	1.73	3
Adams in Y2	1	1.669	ND	2.38										
Adams in Y2	1	6.287		8.36										
					**ALL DATA**	**1.64**	**0.35**	**21**	**20.56**	**11.35**	**10**	**6.28**	**1.59**	**12**

Supplemental Table 1b

Pond/Lake Site Name	Pt #	N2 fix nmol cc h	C fix nmol cc h	Thy pmol cm3 h		N2 Fix nmol cm3 h average	SE	N	C Fix nmol cm3 h average	SE	N	Thy pmol cm3 h average	SE	N
ML1 Y1	1	0.66	122	ND	ML1 Y1	1.31	0.43	9	110	118	7	20.66	3.76	6
ML1 Y1	1	0.45	54	ND										
ML1 Y1	1	1.00	ND	ND										
ML1 Y1	2	2.72	ND	14.94										
ML1 Y1	2	2.76	751	18.62										
ML1 Y1	2	3.41	-244	32.95										
ML1 Y1	3	0.40	40	31.49										
ML1 Y1	3	0.22	-87	12.63										
ML1 Y1	3	0.18	135	13.31										
ML2 Y1	1	1.31	405	ND	ML2 Y1	1.04	0.15	9	350	111	7	36.31	5.46	3
ML2 Y1	1	1.15	968	ND										
ML2 Y1	1	1.04	354	ND										
ML2 Y1	2	1.50	269	ND										
ML2 Y1	2	1.40	104	ND										
ML2 Y1	2	1.50	128	ND										
ML2 Y1	3	0.57	223	40.68										
ML2 Y1	3	0.40	ND	42.79										
ML2 Y1	3	0.51	ND	25.46										
NP Y1	1	2.75	ND	ND	NP Y1	3.55	0.71	9	ND	ND	0	ND	ND	0
NP Y1	1	1.31	ND	ND										
NP Y1	1	1.25	ND	ND										
NP Y1	2	3.42	ND	ND										
NP Y1	2	6.77	ND	ND										
NP Y1	2	7.31	ND	ND										
NP Y1	3	2.92	ND	ND										
NP Y1	3	2.96	ND	ND										

NP Y1	3	3.23	ND	ND										
NP Y2	2	15.38	11146	6.47	NP Y2	10.87	3.44	6	7054	1669	4	7.80	1.67	6
NP Y2	2	23.72	8188	11.70										
NP Y2	2	13.50	ND	14.00										
NP Y2	3	8.69	3508	5.04										
NP Y2	3	1.81	5374	3.78										
NP Y2	3	2.14	ND	5.79										
BL	1	1.67	160	ND	BL	1.27	0.42	9	212	112	9	ND	ND	0
BL	1	0.58	458	ND										
BL	1	1.74	1022	ND										
BL	2	1.08	63	ND										
BL	2	3.47	108	ND										
BL	2	2.76	52	ND										
BL	3	0.00	0	ND										
BL	3	0.00	33	ND										
BL	3	0.10	9	ND										
ML1 Y2	1	0.53	1457	7.08	ML1 Y2	0.76	0.13	6	901	279	4	6.63	0.61	6
ML1 Y2	1	1.05	1302	7.00										
ML1 Y2	1	1.22	ND	7.48										
ML1 Y2	2	0.34	457	5.52										
ML1 Y2	2	0.76	386	8.44										
ML1 Y2	2	0.69	ND	4.28										
HV	1	2.91	3983	ND	HV	1.54	0.43	9	5322	774	9	0.00	0.00	3
HV	1	3.28	2386	ND										
HV	1	1.50	2496	ND										
HV	2	2.68	6227	0.00										
HV	2	1.55	6471	0.00										
HV	2	1.90	5838	0.00										
HV Y2	3	0.01	10017	ND										

HV Y2	3	0	4896	ND										
HV Y2	3	0	5586	ND										
ML2 Y2	1	0.50	ND	ND	ML2 Y2	1.30	0.52	9	ND	ND	0	8.17	2.42	6
ML2 Y2	1	0.62	ND	ND										
ML2 Y2	1	0.59	ND	ND										
ML2 Y2	2	2.86	ND	10.29										
ML2 Y2	2	3.02	ND	11.87										
ML2 Y2	2	4.01	ND	17.23										
ML2 Y2	4	0.08	ND	3.65										
ML2 Y2	4	0.00	ND	2.22										
ML2 Y2	4	0.00	ND	3.77										
					**ALL DATA**	**2.42**	**0.47**	**66**	**2121**	**479**	**40**	**12.28**	**2.11**	**30**

Supplemental Table 1c

Dry Site Name	Pt #	N fix nmol cc h	C fix nmol cc h	Thy pmol cm3 h		N2 Fix nmol cm3 h average	SE	n	C fix nmol cm3 h average	SE	N	Thy pmol cm3 h SE	ND
MS1Y1	4	0.785	0	ND	MS1Y1	0.46	0.16	3	0.00	0.00	3	ND	ND
MS1Y1	4	0.337	0	ND									
MS1Y1	4	0.263	0	ND									
MS2Y1	4	0	0	ND	MS2Y1	0.00	0.00	3	0.00	0.00	3	ND	ND
MS2Y1	4	0	0	ND									
MS2Y1	4	0	0	ND									
ML1	4	0	0	ND	ML1	0.00	0.00	3	1.07	1.07	2	ND	ND
ML1	4	0	2.15	ND									
ML1	4	0	ND	ND									
ML2	4	0	ND	ND	ML2	0.00	0.00	3	ND	ND	0	ND	ND
ML2	4	0	ND	ND									
ML2	4	0	ND	ND									
NP	4	0	ND	ND	NP	0.00	0.00	3	ND	ND	0	ND	ND
NP	4	0	ND	ND									
NP	4	0	ND	ND									
BL	4	0	5.07	ND	BL	0.00	0.00	3	5.07		1	ND	ND
BL	4	0	ND	ND									
BL	4	0	ND	ND									
NP Y2	4	0.012	ND	ND	NP Y2	0.03	0.01	3	ND	ND	0	ND	ND
NP Y2	4	0.044	ND	ND									
NP Y2	4	0.046	ND	ND									

ML 1 Y2	4	0	ND	ND	ML 1 Y2	0.00	0.00	3	ND	ND	0	ND	ND
ML 1 Y2	4	0	ND	ND									
ML 1 Y2	4	0	ND	ND									
AG out	2	0	ND	0.774	AG out	2.30	1.17	3	ND	ND	0	1.16	0.22
AG out	2	3.10	ND	0.928									
AG out	2	3.81	ND	1.766									
ML2 Y2	4	0.08	ND	3.654	ML2 Y2	0.03	0.03	3	ND	ND	0	3.21	0.50
ML2 Y2	4	0	ND	2.220									
ML2 Y2	4	0	ND	3.769									
					**ALL DATA**	**0.28**	**0.16**	**30**	**0.80**	**0.71**	**9**	**2.19**	**0.41**
